# Exploration of High and Low Molecular Weight Polyacrylic Acids and Sodium Polyacrylates as Potential Binder System for Use in Silicon Graphite Anodes

**DOI:** 10.1021/acsaem.4c02672

**Published:** 2025-01-21

**Authors:** Michael J. Jolley, Tanveerkhan S. Pathan, Craig Jenkins, Melanie J. Loveridge

**Affiliations:** Energy Innovation Centre (EIC), WMG, University of Warwick, CV4 7AL Coventry, United Kingdom

**Keywords:** lithium-ion batteries, silicon electrodes, PAA, Na-PAA, molecular weight AC impedance electrode adhesion energy storage application

## Abstract

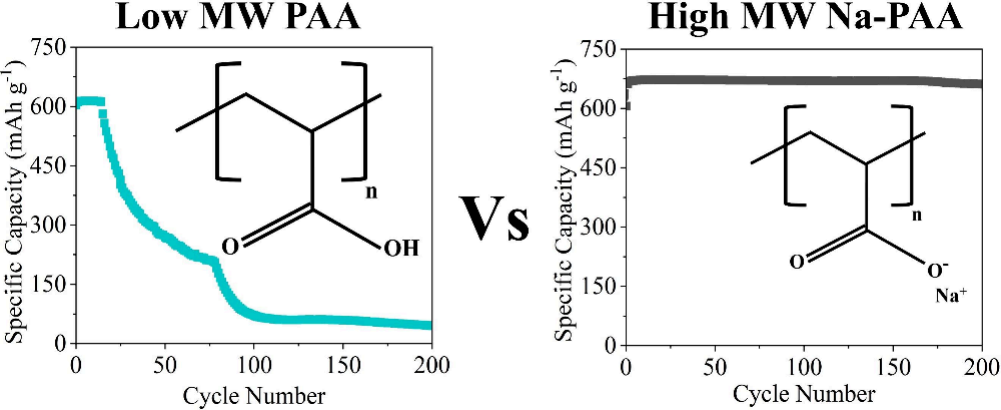

The commercialization of silicon anodes requires polymer binders that are both mechanically robust and electrochemically stable in order to ensure that they can accommodate the volume expansion experienced during cycling. In this study, we examine the use of both low and high molecular weight (MW) polyacrylic acids (PAAs), and sodium polyacrylates (Na-PAAs), at different degrees of partial neutralization, as a possible binder candidate for use in silicon graphite anodes. High MW PAAs were found to have stable capacity retentions of 672 mAh g^–1^ for over 100 cycles, whereas with the low MW PAAs the capacity was found to already have declined to 373 mAh g^–1^ after the first 30 cycles. Furthermore, the partial neutralization of Na-PAA binder system was found to provide superior cycling performances, as compared to non-neutralized or fully neutralized PAA systems. The high MW and partially neutralized PAAs were also found to provide the electrode coatings with higher cohesion strengths, which allow for the electrodes’ microstructure to be more effectively maintained over several cycles. Overall, these findings suggest that partially neutralized and higher MW PAAs are the more suitable polymer binder candidates for use within silicon–graphite anodes.

## Introduction

Silicon remains a promising candidate as the anode active material for the next generation of lithium-ion batteries (LIBs), as it offers several benefits over current commercial graphite anodes.^1^ First silicon has a theoretical capacity of 3579 mAh g^–1^, compared to graphite which is only 372 mAh g^–1^.^2^ Second, silicon has an average working potential of 0.4 V vs Li/Li^+^, whereas for graphite the working potential is 0.25 V vs Li/Li^+^.^3^ Third, silicon is the second most abundant element in the earth’s crust with it being 1500 times more abundant than carbon.^4^ Unfortunately, the commercialization of silicon anodes is hindered due to their undergoing a large volume expansion during cycling, resulting in them becoming cracked and pulverized.^5^ This results in the electrodes experiencing significant capacity fade and short operating lifetimes. Current and traditional polymer binders within the LIBs industry such as polyvinylidene fluoride (PVdF) are inadequate amaintaining the electrode’s microstructure.^6^ The reasoning for this is because PVdF only forms weak van der Waals interactions with the surface of the silicon active material. Therefore, PVdF is inadequate in maintaining the electrode’s microstructure during cycling.^7^

Polyacrylic acid (PAA) and partially neutralized PAA with either sodium hydroxide (NaOH) or lithium hydroxide (LiOH) to produce sodium/lithium polyacrylate (Na-PAA/Li-PAA) has attracted a great deal of attention in the battery field, as a binder system for both graphite and silicon anodes.^6,8−10^ This is due to it being nontoxic, water-soluble, and containing a significant number of carboxylate groups, which form strong covalent interactions with the surface of the active material particles.^8^. In addition to this, it has also been found that partial neutralization of PAA with sodium ions can lead to an even more effective binder system for silicon anodes.^11^ This is due to the polymer chains transforming from an agglomerated state in PAA to an extended configuration in Na-PAA or Li-PAA, resulting in a better distribution of the binder system within the electrode coating.^8^

Though the MW of PAA ranges between 3 and 1250 kDa, the majority of the battery industry focuses on using PAAs with high MWs between 400 and 450 kDa PAA.^12−14^ A rare exception to this is the study by Kasinathan et al., who explored the possibility of using an ultrahigh 1250 kDa MW PAA as the binder system for silicon/graphite anodes, and compared it with standard high MW 250 and 450 kDa PAAs.^15^ Kasinathan et al. concluded that ultrahigh MW PAAs are not suitable for the application of binder systems, as their high viscosities make the electrode slurry far too viscous for processing during the electrode manufacturing stage. Instead they recommended using medium 250 and 400 kDa MW PAA systems, as these binders were found to provide high adhesion strength within the electrodes and optimal rheological properties for the electrode slurries during the electrode manufacturing process.

However, 250 and 450 kDa MW PAA polymers possess glass transition temperatures (*T*_g_) values that are often above the operating temperatures of LIBs, meaning they display stiff or brittle polymer behaviors when operating.^16^ As a result, these higher *T*_g_ values make high Mw PAA polymers unsuitable for dealing with the strain experienced during the volume expansion and to overcome this, the capacity of the anode has to be limited.^17^ A possible solution to this issue is to use lower MW PAA and Na-PAAs instead, as these typically possess lower *T*_g_ values and would display more elastic and flexible characteristics when operating in LIBs.^18^ This would allow the polymer binder to demonstrate more elastic behavior, that could more easily buffer the strain of the volume expansion and as such more effectively maintain the electrodes’ microstructure during cycling.

Low MW PAAs have previously been investigated for the application of silicon/graphite anodes by B. Hu et al., who explored the use of Li-PAA’s ranging from 3 to 150 kDa.^19^ B. Hu et al. reported that Li-PAAs with MWs between 24 000 and 150 000 g mol^–1^ make suitable binder candidates, while suggesting that low MW Li-PAAs with MWs of ≤24 000 were unsuitable, because they provide insufficient cohesion/adhesion strengths necessary to maintain the electrodes microstructure during cycling.^19^ Low MW Li-PAAs have also been used as a dispersant in conjunction with high MW Li-PAAs for the application of silicon/graphite anodes as reported by B. L. Armstrong et al.^20^

While low MW Li-PAAs have been explored for application as the binder system for silicon/graphite anodes, to the best of our knowledge, low MW Na-PAAs have not. This is potentially an important omission, as it has been reported that the neutralization of PAA by NaOH compared with that of LiOH results in the carboxylate groups being more effectively dissociated within the polymer chains. In addition to this, the sodium counterion from Na-PAA is more efficient at improving the solid electrolyte interphase (SEI) layer properties, resulting in a superior battery performance.^21^ Therefore, the use of low MW Na-PAAs could combine these benefits with the ones associated with low MW polymers outlined above. Here in this study, we investigate the use of low MW Na-PAA at different degrees of neutralization as a possible binder system for silicon graphite anodes. We also compare their performances directly to their corresponding high MW PAA counterparts.

## Experimental Methods

### Preparation of PAA Binders

For PAA-1 and PAA-2, 12 g of PAA (Sigma-Aldrich, purity ≥99.5%, 450 000 MW) was added slowly to 88 mL of distilled water. The solution was mixed with an overhead high-speed homodisperser at a speed of 1000 rpm for 1 h, followed by degassing at ambient conditions for 24 h. For PAA-2 4.73 g of NaOH (Sigma-Aldrich, ≥99.5%, 40 g mol^–1^) was dissolved in 25.6 mL of distilled water, and then added slowly to the PAA solution on stirring pad (IKA RCT basic IKAMAG safety control magnetic stirrer), to produce a 70% partially neutralized Na-PAA binder system. The binder solution was left for a further 24 h for degassing. The final solid content of the binder was found to be 14 wt %, measured with a moisture analyzer (Ohaus MB120). The resultant pH of the solution was tested with pH indicator strips (VWR BOH chemicals) and found to be between pH 6 and pH 7. PAA-3, PAA-4, and PAA-5 were all supplied directly from Synthomer without any further modification. Table 1 provides an overview of the different PAAs used in this study.

**Table 1:** Overview of the Different Polymer Binders

product	MW	PAA	pH	solid content (%)	degree of partial neutralization (%)
PAA-1	high	PAA	2.0–3.0	12	0
PAA-2	high	Na-PAA	6.0–7.0	12	70
PAA-3	low	PAA	2.0–3.0	53	0
PAA-4	low	Na-PAA	8.0–9.0	42	100
PAA-5	low	Na-PAA	6.3–7.3	35	80

### Electrode Fabrication

To ensure that all the electrodes had a final formulation with a dry mass % composition of 30:50:10:10 (silicon: graphite:conductive additive:binder), the amount of PAA binder added varied between the slurries. This is due to the different solid contents of the PAA binder systems, as shown in Table 1. For example, to ensure that the slurries contained 3 g of PAA solid, 25 g of the 12% PAA-1 binder system was added, whereas only 5.76 g of the 53% PAA-3 binder system was added. Table 2 provides an overview of the electrode slurry formulations used in this study. An example of the electrode manufacturing process is outlined below.

**Table 2:** Overview of Electrode Slurries Prepared in This Study

electrode slurry	Si (g)	graphite (g)	C-45 (g)	PAA (g)
PAA-1	9	15	3	25
PAA-2	9	15	3	25
PAA-3	9	15	3	5.76
PAA-4	9	15	3	7.21
PAA-5	9	15	3	8.57

3 g of carbon black, 15 g of graphite (BTR FC-18), and 25 g of the12% PAA-1 system (for PAA-2, PAA-3, PAA-4, and PAA-5; see Table 2) were added to 20 mL of water and initially mixed by hand with a spatula. The mixture was placed on an overhead high-speed homodisperser at 620 rpm for an hour, before being placed under an ultrasonic probe (UP400S, SciMED) at 0.5 cycles, 65% amplitude for 15 min. 9 g of 0.02–2000 μm sized silicon powder (E-410 Elkem) was added, and the mixture was placed back under the ultrasonic probe at 0.5 cycles, 65% amplitude for 3 min. The mixture was dispersed once more using the overhead high-speed homodisperser at 1000 rpm for a further 30 min. 15 g of 12% PAA binder was added, and the slurry was mixed for a further 30 min at 1000 rpm. The slurry mixture was transferred to a Filmix mixing vessel (Filmix model 40-L PRIMIX) and subjected to the final stages of mixing of 30 s at 10 ms^–1^ and 30 s at 25 ms^–1^. The resultant mixture was transferred to a 300 mL high-density polyethylene (HDPE) thick-walled jar (Intertreon). The resultant slurries were allowed to degas for 30 min.

The electrode slurries were coated onto 11 μm thick copper foil (Oak Mitsui, electrodeposited) by using a draw-down coater (RK Instruments Ltd.) at a blade-gap of 90 μm. Coatings were placed on a hot plate at 50 °C to remove the majority of the solvent, before being stored in a vacuum oven at 50 °C for a period of 24 h. The above mixing procedure results in electrode formulation with a dry mass % composition of 30:50:10:10 (silicon:graphite:conductive additive:binder). Table 2 provides an overview of the electrode slurry formulations used in this study.

### Viscosity

The viscosities of the different PAA polymer binder systems were measured at 25 °C using a rotational rheometer (Rheolab QC Anton Paar). Measurements were taken using a C-CC14 concentric cylinder measuring system and disposable cup, with continuous shear: a rate sweep measurement between shear rates of 0.1 s^–1^ and 1000 s^–1^

### Electrochemical Characterization

CR2032 coin cell kits (Hohsen Corporation) were used to assemble anode half-cells vs lithium. Electrode coatings were dried in a vacuum oven (Binder-VD 53 vacuum drying oven with integrated vacuum pump system) at 50 °C for period of 24 h. Electrodes were cut out using a 15.0 mm electrode cutter (supplier Zhengzhou CY Scientific Instrument Co, Ltd.) and weighed out on a microbalance (Sartorius). Precut 15.6 mm × 0.25 mm thick lithium discs (Pi-Kem Limited) were used as counter electrodes and were stored under vacuum. The surface of the lithium was cleaned with a toothbrush (Wisdom Smokers Extra Hard Toothbrush) to remove the native oxide layer. The separator used was a trilayer polypropylene–polyethylene–polypropylene (Celgard 2325), which was cut into 19 mm diameter discs. All cell components were dried in a vacuum oven for 24 h before being assembled into cells. Commercial RD265 electrolyte (Soulbrain), containing 1.2 M LiPF_6_ in EC/EMC 1/3 v/v + 3% wt VC + 15% FEC, was used for the silicon/graphite system. The electrolyte systems were stored in an argon-filled glovebox, with small samples being removed for cell making.

Silicon/graphite anode half-cells (vs Li/Li^+^) were tested using a Maccor series cycler using a constant current (CC) method in a 25 °C temperature-controlled oven. For the formation cycle, the cells were discharged to 5 mV and then charged back up to 1.0 V, at a C rate of C/10. Further discharge/charge cycling was carried out at a C rate of C/2, between these voltage limits.

**Figure 1:**
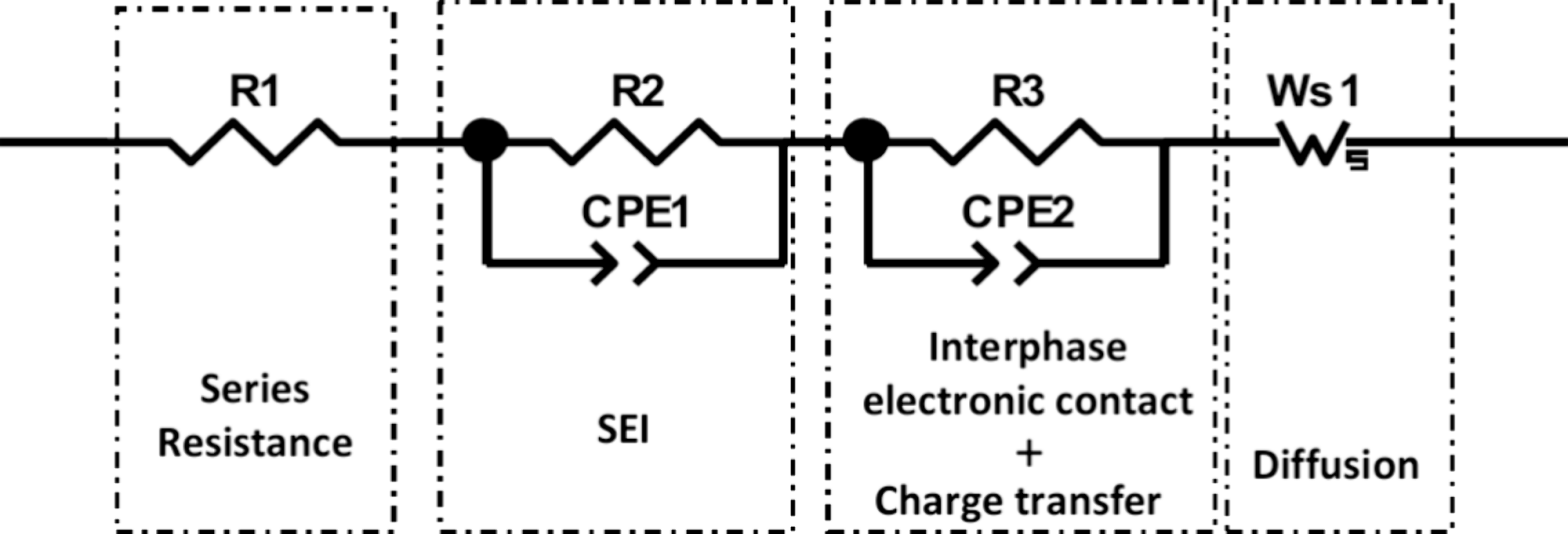
Equivalent circuit model used for fitting EIS data.

Potentio electrochemical impedance spectroscopy (PEIS) was carried out using a VMP3 potentiostat (Bio-Logic), for measuring the impedance change as a function of cycle number. The test was conducted with voltage amplitude of 10 mV, measured between frequencies of 500 kHz and 100 mHz at 50% state of charge (SoC) of each cell. The first measurement was taken after the formation cycle with additional 10 min relaxation time and repeated every 10 cycles. ZView software was used for the impedance fitting using the equivalent circuit model as outlined in Figure 1.

### Rate Capability Tests

To investigate how the binder systems could cope with fast charging, the cells were charged for 5 cycles at the following C-rates: C/20, C/10, C/5, C/2, 1C, 2C, 5C, 10C, and C/20.

### Adhesion/Cohesion Electrode Coating Testing

The adhesion/cohesion strength of electrode coatings were determined by a compression/tensile adhesion test using a Zwick Roell 0.5 kN tensile tester with a Xforce P load cell, capacity 500 N, and *Z*-direction tensile test adaptor. A layer of double-sided polyacrylate tape on a polypropylene substrate (5696, Tesa) was placed on the lower specimen carrier. A 2 kg standard manual adhesive test pressure roller was used to ensure that the tapes were firmly stuck to the specimen. An electrode coating sample, which was slightly larger than the specimen carrier, was placed on top of the layer of tape with the coating side facing upward. A scalpel blade was used to remove the excess electrode coating which overhung the specimen carrier. Electrode coating samples were divided into 625 mm^2^ specimens, following the predetermined cutting guidelines on the specimen carriers.

Samples of double-sided tape (1.13 cm^2^) were cut and placed on the upper specimen plate. The 2 kg roller was again used to ensure the tape was firmly stuck onto the surface of the specimen carrier. Samples were first compressed to a compression of 600 kPa at a velocity of 0.75 mm min^–1^ and were held under this compression for 30 s. Afterward the upper plate was pulled off at a velocity of 100 mm min^–1^ and the maximum tensile strength was recorded at a data acquisition rate of 2 kHz.

### Electrolyte Swelling Tests

Polymer film samples were prepared by placing a polypropylene gasket casing coin cell part on a Teflon release sheet. The gasket was filled with 1–2 mL of polymer, and samples were then allowed to air-dry for 24 h. The samples were then placed in an oven at 60 °C overnight for further drying, after which the Teflon release sheet and gasket were carefully removed. The samples were then weighed and placed back in the oven for further drying. Samples were reweighed until a constant mass was obtained.

PAA polymer samples were placed in a 300 mL HDPE thick-walled jar and dried in an oven at 60 °C overnight before being transferred to an argon filled glovebox. Polymer samples were weighed using an analytical balance, before being immersed in 5 mL of commercial RD265 electrolyte for 24 h. After 24 h, the polymer/electrolyte system was filtered to remove the polymer samples from the electrolyte. Excess electrolyte was wiped off from the surface sample with a tissue, and the sample was weighed, taped, dried, and reweighed.

### Fourier Infrared Spectroscopy (FTIR)

FTIR was conducted using an FTIR spectrometer (Vertex 70) and was run on polymer films for all the samples. The resolution setting was 4 cm^–1^ with 80 scans from the wavenumber range of 4500–500 cm^–1^.

### Tensile Testing

Polymer film samples of PAA-2 and PAA-5 were developed by pouring 10 mL of the solution into a precut Teflon dish (Craignator 3000 Gilbert Curry Industrial Plastics Co., Ltd.). Samples were air-dried for 24 h before being removed from the Teflon dish, weighed, and placed back on the Teflon sheet for further air drying. This was continued until a constant mass was obtained. Polymer films with the thickness of 0.25–0.45 mm were cut into tensile specimens according to ASTM D638, with a gauge length of 26 mm and a width of 3.26 mm. Tensile testing was performed using a 100 kN Instron tensile tester, 1 kN load cell, wedge grips, with a video extensometer, and at an extension rate of 5 mm min^–1^. Young’s modulus was calculated using a chord modulus fitted between 0.7% and 1.5% offset strain.

## Results and Discussion

### Polymer Characterization FTIR

**Figure 2:**
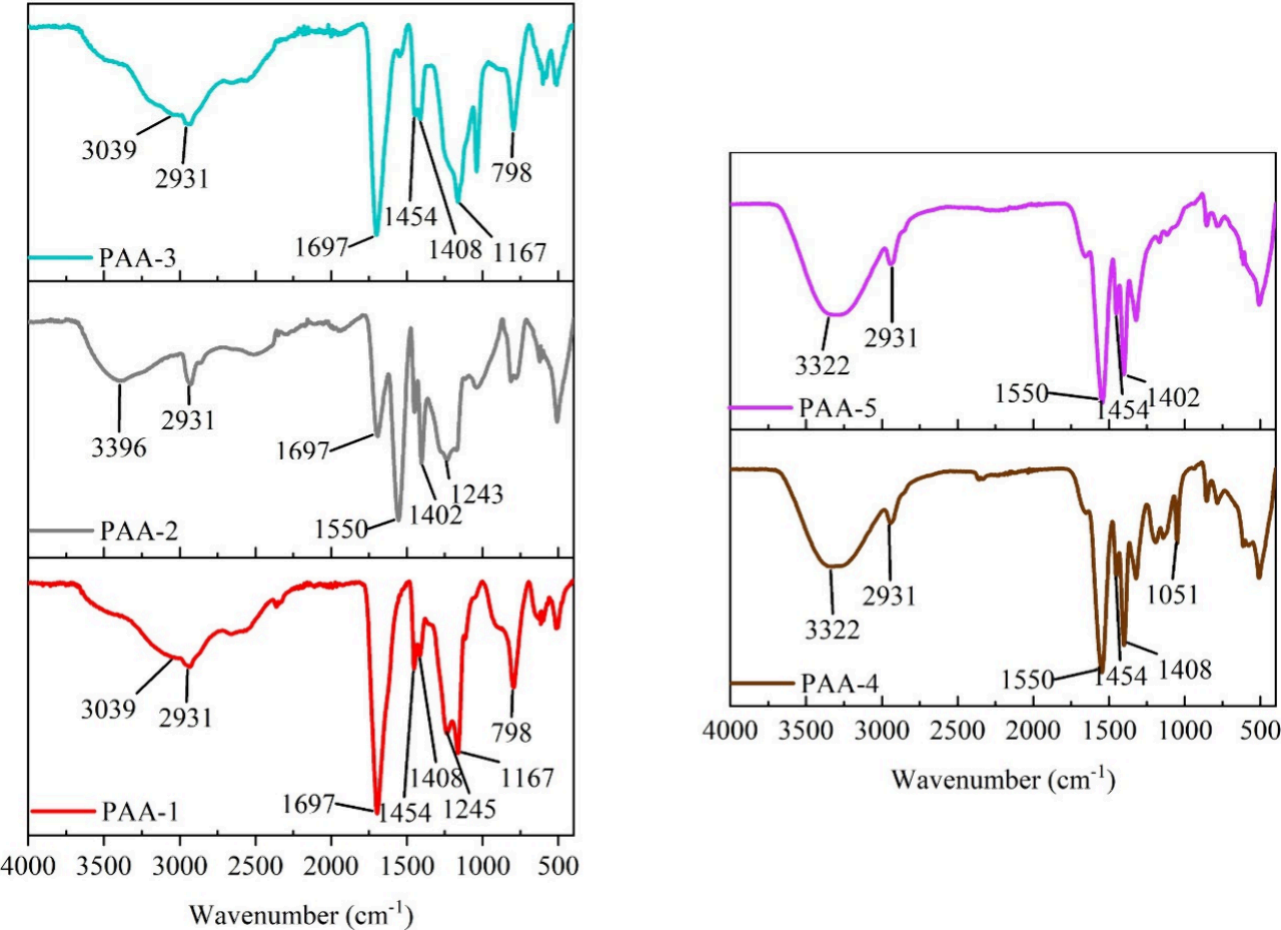
FTIR spectra of the different PAA binder systems.

Polymer films were prepared for the different PAAs and their chemical structures were characterized by FTIR. The resultant spectra are displayed in Figure 2. Both the non-neutralized PAA samples (PAA-1 and PAA-3) showed a broad peak at 3039 cm^–1^, which is due to the O–H stretch from the carboxylic acid functional group. This overlaps with the peak at 2931 cm^–1^ which is a result of C–H stretching in CH_2_ groups along the backbone chains. The sharp peak at 1697 cm^–1^ is due to the carbonyl (C=O), while the medium sharp peak at 1454 cm^–1^ is a result of CH_2_ asymmetric stretching. Peaks at 1408, 1243, and 1167 cm^–1^, are due to the in plane C–O–H bonding mode in PAA, as well as the symmetric and asymmetric stretching of the C–O bond, respectively. The final peak at 798 cm^–1^ is a result of the out of plane OH···O deformation. It is reported that this peak is an indication of strong hydrogen bonding present between the polymer chains, most likely due to their agglomeration.^22^

Several differences can be observed in the spectra for PAA-2, PAA-4, and PAA-5. First, the O–H peak is more distinctive having shifted to a higher wavenumber of 3322 cm^–1^ and is no longer overlapping with the CH_2_ peak. This shift is reported to be related to the distribution and to a decrease in strength of the hydrogen bonding within the polymer chains, suggesting that the polymer chains in PAA-2, PAA-4 and PAA-5 are now in an extended configuration.^23^ For PAA-4 and PAA-5 the carbonyl peak has completely shifted to 1550 cm^–1^ due to the carboxylate ion being formed during the neutralization. 1550 and 1402 cm^–1^, are due to the asymmetric and symmetric stretching of COO–.^22^ Meanwhile PAA-2 also displayed these carbonyl peaks, as well as the original peak at 1697 cm^–1^, due to there being a remaining 30% of non-neutralized COOH groups present.

### Polymer Viscosity

**Figure 3:**
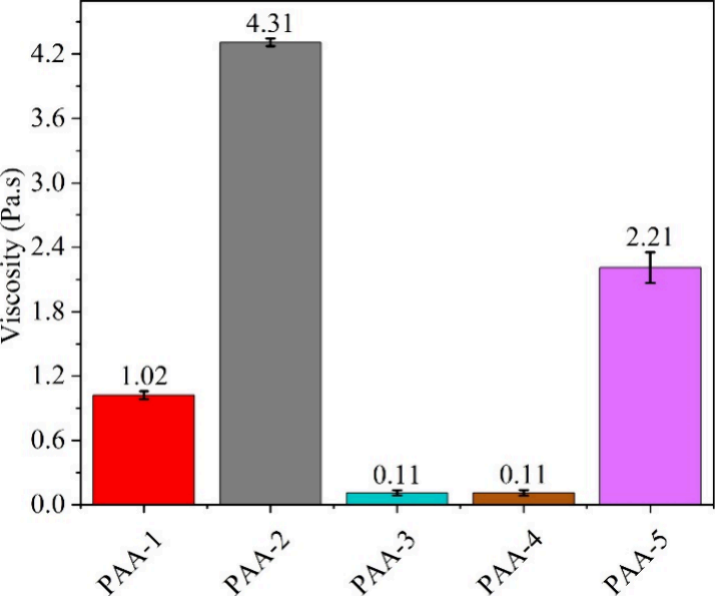
Viscosities of aqueous PAA polymer systems at a shear rate of 10 s^–1^.

A sweeping shear rate between 0.1 to 1000 s^–1^ was used to measure the viscosities of all the PAA binder systems, and their viscosities at a shear rate of 10 s^–1^ are provided in Figure 3. Most of the low Mw PAAs were found to have significantly lower viscosities compared with the high MW PAAs. For example, viscosity of the low MW non-neutralized PAA-3 was 0.11 Pa·s, whereas its ats high MW counterpart (PAA-1) was 1.02 Pa.s. This is due to the longer polymer chains present in the PAA-1, having a greater number of interactions with the water molecules as well as other neighboring polymer chains. This results in heavily restricting the mobility of water molecules causing the viscosities of the polymer to be increased.^24^

The viscosity of the high MW partially neutralized Na-PAA, PAA-2 system was found to be 4.31 Pa·s, which is even higher than PAA-1. The same trend applies to the low MW systems, PAA-3 and PAA-5, where the viscosities were 0.11 and 2.21 Pa·s, respectively. This is due to the polymer chains in PAA-1 and PAA-3, agglomerating together through hydrogen bonding, whereas in PAA-2 and PAA-5 the chains are in an extended configuration from electronic repulsions of their negatively charged carboxylate groups.^8^ However, the viscosity of the fully neutralized low MW PAA system (PAA-4) had a viscosity measurement of 0.11 Pa·s, which is similar to that of PAA-3. The decrease in viscosity of the fully neutralized PAA system is due to a process known as “*the charge screening effect*”. Here the presence of an excess amount of counter cations shields the negatively charged carboxylic groups, hindering the electronic repulsions between the polymer chains.^25^ This then causes a decrease in the viscosity because it hinders the polymer chain’s configuration, limiting it is ability to restrict the mobility of the water molecules.^26^

These differences in viscosities between the PAAs were found to directly influence the corresponding slurries during the electrode manufacturing process. For example, the slurries containing the low viscosity polymers (PAA- 3 and PAA-4) were found to be very runny compared to the slurries containing PAA-1, PAA-2, and PAA-5. A direct consequence of this is observed in the electrode mass loadings in Table 3, where PAA-3 and PAA-4 are shown to have lower mass loadings compared to the other PAA systems. First, the lower viscosities of these polymers make the slurry runnier, giving it a greater chance to spread faster and nonuniformly over the current collector during the coating process.^27^ Second, in electrode slurries with lower viscosities, there is a greater chance of sedimentation occurring within the slurry, because the kinetic energy of the particles within the slurry is higher. Therefore, there is a greater chance of them overcoming the activation barrier required for the particle sedimentation process, resulting in a slurry being casted with a lower solid content than expected.^28^

**Table 3:** Overview of the Electrode Mass Loadings Tensile Testing^a^

binder system	electrode mass loadings (mg cm^–2^)
PAA-1	3.290 ± 0.042
PAA-2	2.907 ± 0.015
PAA-3	2.129 ± 0.330
PAA-4	2.306 ± 0.229
PAA-5	3.399 ± 0.022

a ± refers to the standard deviation based on a minimum of five specimens.

### Electrochemical Characterization

**Figure 4:**
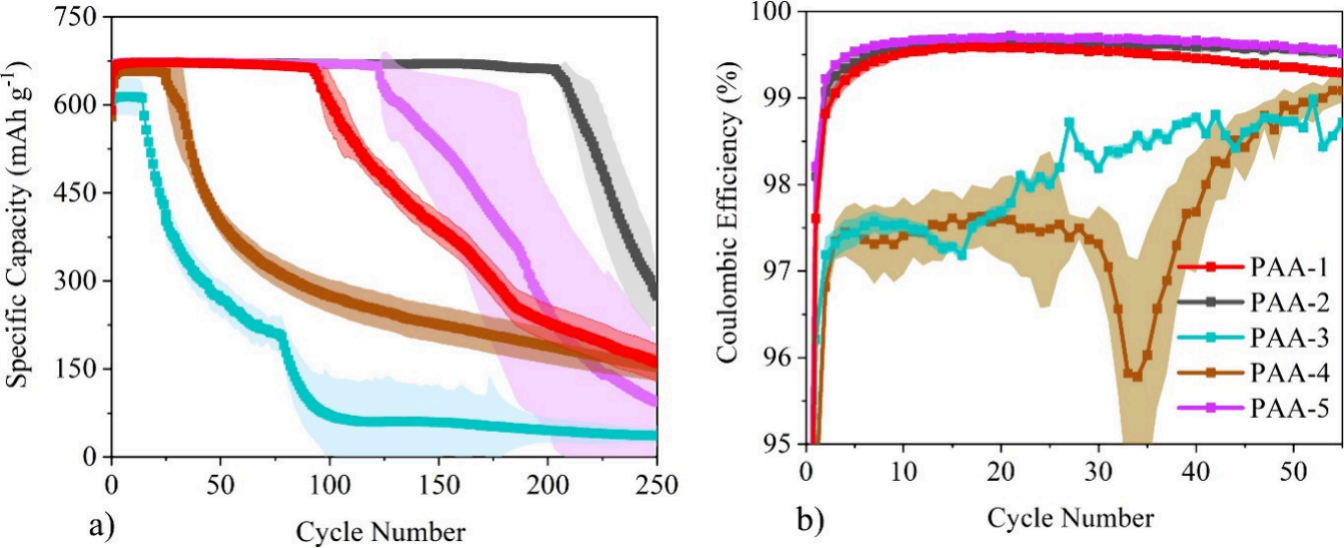
(a) Cycling performance for all PAA binders in silicon/graphite anode half-cells for the first 250 cycles at C/2 and (b) Coulombic efficiency for the first 50 cycles.

The charge–discharge cycling performances of all of the PAA systems are provided in Figure 4a. Three cells were made for each of the PAA systems, and the standard deviation is plotted as the error bar area. For all the PAA systems, an initial rise in capacity was observed over the first few cycles. This is attributed to the silicon going through an activation process of being converted from a crystalline to an amorphous phase, which is a more favorable environment for lithium-ion diffusion kinetics.^1^ All the systems then have a stable capacity retention before experiencing a significant decline, commonly acknowledged as the “*rollover effect*” and was found to occur at very different points for each of the PAA systems.^29^ The capacity retention for the low MW PAA-3 was stable for 20 cycles, whereas for the high MW PAA-1 it was stable for 100 cycles. This improved stability in PAA-1 is due to the longer polymer chains found within the higher MW PAA, therefore allowing for there to be more points of interaction between the polymer binder and active material. This therefore allows for enhanced cohesion strength within the electrode coating and as such for a greater electrode microstructure stability^30^. Meanwhile, the capacity retention of PAA-2 (high MW partially neutralized Na-PAA), was found to exceed that of PAA-1, and was stable for over 200 cycles. This is due to the partial neutralization prohibiting the polymer chains from agglomerating, as the negatively charged carboxylate groups cause electronic repulsion. As a result there is an improved distribution of the polymer binder throughout the electrode coating, which therefore increases the amount of interactions between the active material and polymer binder.^8^

A similar trend was found with the neutralized and non-neutralized low MW PAA samples with the capacity retention, PAA-3, PAA-4, and PAA-5 having stable capacity retentions for 20, 45, and 120 cycles, respectively. Meanwhile, the significant difference in performance between Na-PAA systems (PAA-4 and PAA-5) can be attributed to their different degrees of neutralization. PAA-4 is a fully neutralized Na-PAA system, whereas PAA-5 is an 80% partially neutralized Na-PAA system. The shorter capacity retention of PAA-4 again is most likely a result of the charge screening effect.^25^

PAA-1, PAA-2, and PAA-5 were also found to demonstrate more stable Coulombic efficiencies (CE) values in Figure 4b compared to PAA-3, and PAA-4. The CE values for the different binder systems were calculated by eq 1, and the values for cycles 1, 5, 20, and 50 are provided in Table 4.
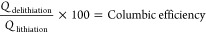
1where *Q*_delithiation_ is the delithiation capacity (charge capacity in anode half-cells) and *Q*_lithiation_ is the lithiation capacity (discharge capacity in anode half-cells).

**Table 4:** Overview of Coulombic Efficiency for Different PAAs at Cycles 1, 5, 20, and 50^a^

binder system	1st cycle (%)	5th cycle (%)	20th cycle (%)	50th cycle (%)
PAA-1	87.52 ± 0.20	99.50 ± 0.09	99.59 ± 0.05	99.36 ± 0.05
PAA-2	89.76 ± 0.24	99.40 ± 0.16	99.63 ± 0.08	99.57 ± 0.06
PAA-3	89.44 ± 0.34	97.44 ± 0.19	97.69 ± 0.07	98.73 ± 0.04
PAA-4	85.81 ± 0.13	97.44 ± 0.35	97.60 ± 0.48	98.86 ± 0.19
PAA-5	90.05 ± 0.16	99.54 ± 0.02	99.70 ± 0.02	99.6 ± 0.04

a ± refers to the standard deviation based on three cells.

The first cycles CE values of the cells ranged between 85–91%, which is due to the formation of the SEI layer, which results in the consumption of lithium ions and an irreversible capacity loss.^31^ In a commercial full cell, only a limited reservoir of lithium is available in the cell, and thus, any lithium that is irreversibly consumed during the formation cycle reduces the overall amount of energy stored within it. However, the work carried out in this study is based on silicon graphite anode half-cells where the counter electrode is a pure lithium disc, and thus an excess of lithium is available.^32^

After the first cycle, the CE values for the PAA-5, PAA-1, and PAA-2 systems quickly rose to over 99.5%, suggesting that for these systems the surface of the silicon active material was fully covered by the SEI layer.^33^ Higher CE values are also linked to less lithium being irreversibly consumed and improved cycle lifetimes, agreeing with the cycling data in Figure 4a.^34^ Meanwhile, the CE values for PAA-3 and PAA-4 only reached between 95 and 97% and were found to decline during the first 30 cycles, indicating that further side reactions are occurring with the electrolyte to produce a fresh SEI layer. This suggests that the silicon is no longer fully covered by the SEI layer, with fresh surfaces of the active material becoming exposed. If these binder systems are unable to maintain the electrode’s microstructure during the volume expansion, this will result in cracking and exposing fresh surfaces of active material.^35^ The higher CE values for the PAA-5, PAA-1, and PAA-2 systems suggest that these systems are better at assisting in the formation of a stable SEI layer on the surface of the active material.^36^

For PAA-2 and PAA-5, this can be explained through the extended configuration of their polymer chains which results in there being a thin homogeneous layer of binder coated on the surface of the silicon and graphite active material particles during the electrode manufacturing process.^37^ This layer of binder acts as an artificial SEI layer, which then minimizes the direct contact between the active material particles and electrolyte, hence reducing the SEI formation process.^38^ Meanwhile, the agglomerated nature of the polymer chains in PAA-3 and PAA-4 instead results in a heterogeneous distribution of pockets of binder over the surface of the active material particles. As the binder is not homogeneously distributed, there is a slighter greater amount of direct contact between the active material and electrolyte.^37^ Finally, the PAA-1 system displays high CE values, even though its polymer chains are also in an agglomerated state and should therefore have pockets of binder heterogeneously distributed over the surface of the particles. However, due to its high MW, there is an increase in the amount of polymer present on the surface of the silicon particles, which again then minimizing the direct contact between the particles and electrolytes.^39^

For PAA-3 and PAA-4 the CE values for these binder systems were found to increase again after their sharp decline at 30 cycles, while their capacities continued to fade. In this study, the theory is that the CE can remain high even if the discharge capacity declines, but only if the charge capacity also declines. This is because if the discharge and charge capacities were low, the ratio between the two would be high, hence a high CE value.

**Figure 5:**
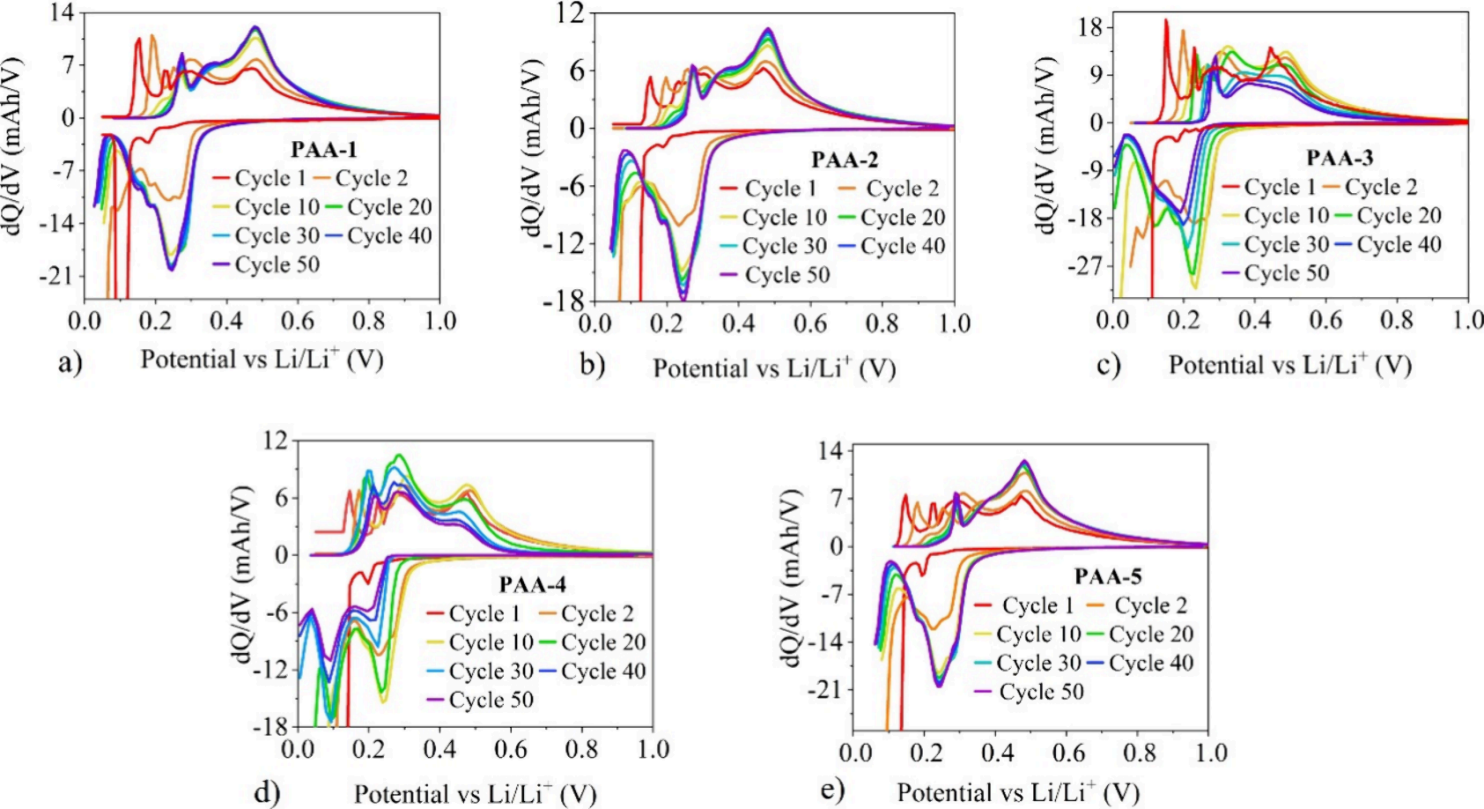
d*Q*/d*V* vs voltage plots for (a) PAA-1, (b) PAA-2, (c) PAA-3, (d) PAA-4, and (e) PAA-5.

The d*Q*/d*V* vs voltage plots for the different binder systems at cycles 1, 2, 10, 20, 30, 40, and 50 are provided in Figure 5. A cathodic discharge peak at 0.1 V was observed in all first cycle d*Q*/d*V* profiles, which is associated with the lithiation of crystalline silicon.^5^ Meanwhile, the observed small shoulder peaks at around 0.2 V occur due to the lithium graphite intercalation, as this is known to occur at voltages between 0.07 and 0.20 V.^40^ During the delithiation, the peaks around 0.28 and 0.47 V are attributed to the phase conversions of a-Li_3.5_Si → a-Li_2_Si and a-Li_2_Si → a-Si, respectively.^41^ The peaks between 0.11 and 0.23 V are attributed to the deintercalation of graphite (Li_*x*_C_6_ → C_6_ + *x*Li^+^ + *x*e^–^).^2^

Significant differences were observed in the d*Q*/d*V* plots from cycle 2 onward, strongly indicating that the different PAA binder systems affect the interfacial reactions between the silicon and lithium ions.^42^ In the second cycle d*Q*/d*V* plots, all of the binder systems displayed a cathodic shoulder peak at 0.30 V and a strong peak at 0.23 V, which corresponds to the a-Si → a-Li_2_Si phase conversion. They also had a peak at 0.08 V, which is due to the a-Li_2_Si → a-Li_3.5_Si phase conversion, instead of only having a single peak at 0.1 V.^43^ The presence of these peaks confirm that the phase conversion from crystalline to amorphous silicon was successful.^42^

Differences between the first and second cycle d*Q*/d*V* plots are observed because the lithiation of crystalline silicon undergoes an mechanism alternative to that of amorphous silicon. In the initial lithiation cycle the conversion of crystalline to amorphous silicon requires an activation barrier to be overcome, which is not required in later cycles.^44^ However, both the PAA-1 and PAA-3 systems still displayed a 0.1 V peak in their second cycle d*Q*/d*V* plots. This occurs if the crystalline silicon has a relatively large particle size and cannot be fully converted to the amorphous silicon during the first cycle. In both systems, the intensity of this peak decreases with further cycling and eventually disappears as more of the electrode passes through the activation process.^45^

Between cycles 2 and 10, the lithiation peaks in the d*Q*/d*V* plots for all of the systems shifted to lower voltages, while the delithiation peaks shifted to higher voltages. This shifting is due to the thickening of the SEI layer, and can result in an increase in resistance, and hence increased polarization.^46^ Significant differences can be observed between the d*Q*/d*V* plots for cycles 10–50 for the PAA-3 and PAA-4, compared with the PAA-5, PAA-1, and PAA-2. Between the 10th and 50th cycle the peaks in the PAA-5, PAA-1, and PAA-2 d*Q*/d*V* plots displayed no further shifting in the voltage and only a slight alteration to their intensities. On the contrary, by the 50th cycle, the discharge peaks had merged and experienced a dramatic decline in intensity in the PAA-3 and PAA-4 systems. These decreases in peak intensities highlights that these binder systems provide a lack of stable electrochemical reversibility.^47^ In PAA-4, the intensity of the shoulder peak at 0.30 V declined over the cycles and by the 50th cycle, it had completely disappeared. The disappearance of this peak indicates that their capacity declines could be due to incomplete lithiation of the silicon active material, as these binder systems provide a less favorable environment for lithium interaction.^48^ Again, this can be attributed to the PAA-1, PAA-2, and PAA-5 systems forming a more homogeneous artificial SEI layer over the particles of the active materials compared to the PAA-3 and PAA-4 systems.^37^ This is significant as lithium-ion intercalation kinetics is highly determined by the SEI layer, as all lithium ions have to transport through it before they undergo a desolvation process. The presence of carbonyl groups within the SEI layer has been reported to enhance this desolvation step for the removal of lithium ions from EC solvent molecules within the electrolyte.^38^ PAA-3 and PAA-4 provide a less favorable environment, as the nonhomogenous layer of their binders results in there being less direct contact between the artificial SEI layer and the EC solvated lithium ions so the desolvation process is not as enhanced to the same degree as it is in the other systems.

**Figure 6:**
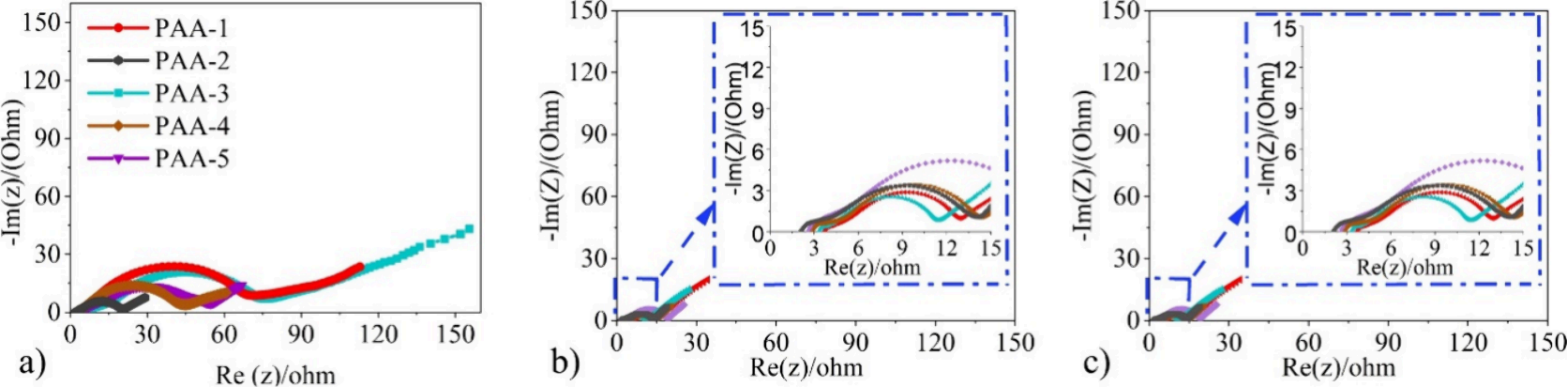
EIS Nyquist plots for 50% SOC at (a) cycle 2, (b) cycle 10, and (c) cycle 70.

As stated above, all the PAA systems in Figure 4a demonstrated behavior referred to as the “*roll over effect*”, and one of the factors that causes this is the thickening of the SEI layer.^29^ As an increase in impedance resistance has been linked to an excess SEI layer growth, EIS measurements were performed on the different binder systems to further investigate this behavior.^49^Figure 6 provides the Nyquist plots taken at 50% SOC during (a) cycle 2, (b) cycle 10, and (c) cycle 70 for all the PAA binder systems. A typical Nyquist plot for LIBs should consist of two semi circles and an angled line, relating to the series resistances (*R*_SEI_), charge transfer resistance (*R*_CT_), and Warburg impedance (*Z*_w_), respectively. In Figure 6a, remarkable differences between the second cycle Nyquist plots were observed. First, the semi circles in cycle 2 for the PAA-1 and PAA-3 systems were significantly larger than the semicircles from the PAA-5 and PAA-2 systems. Larger semi circles are due to a more loosely packed electrode with inferior lithium-ion transport efficiencies that arise if there is a weaker degree of hydrogen bonding interactions present between the polymer binder and active material.^9^ This is likely to be the case with the PAA-3 and PAA-1 systems because their carboxylate groups will primarily be in the protonated state. Therefore, their polymer chains will agglomerate instead of form interactions with the active material.

With the PAA-5 and PAA-2 systems, the neutralization causes the carboxylate groups to primarily be in the dissociated state. Because of this, these binder systems form more hydrogen bonding interactions with the active material, producing a more densely packed electrode. Hence these systems displayed smaller semi circles in their second cycle Nyquist plots. The magnitudes of the semicircles from the Nyquist plots of all the systems significantly decrease by cycle 10 (Figure 6b). This is again due to the silicon undergoing an initial activation process of being converted from crystalline to an amorphous state.^50^ Between cycles 10 and 70, the magnitudes of the semi circles for the PAA-3 and PAA-4 were found to increase. Meanwhile, the semicircles for PAA-5, PAA-1 and PAA-2 remained relatively constant between cycles 10–70, indicating that the levels of resistances for these systems remained constant.

**Figure 7:**
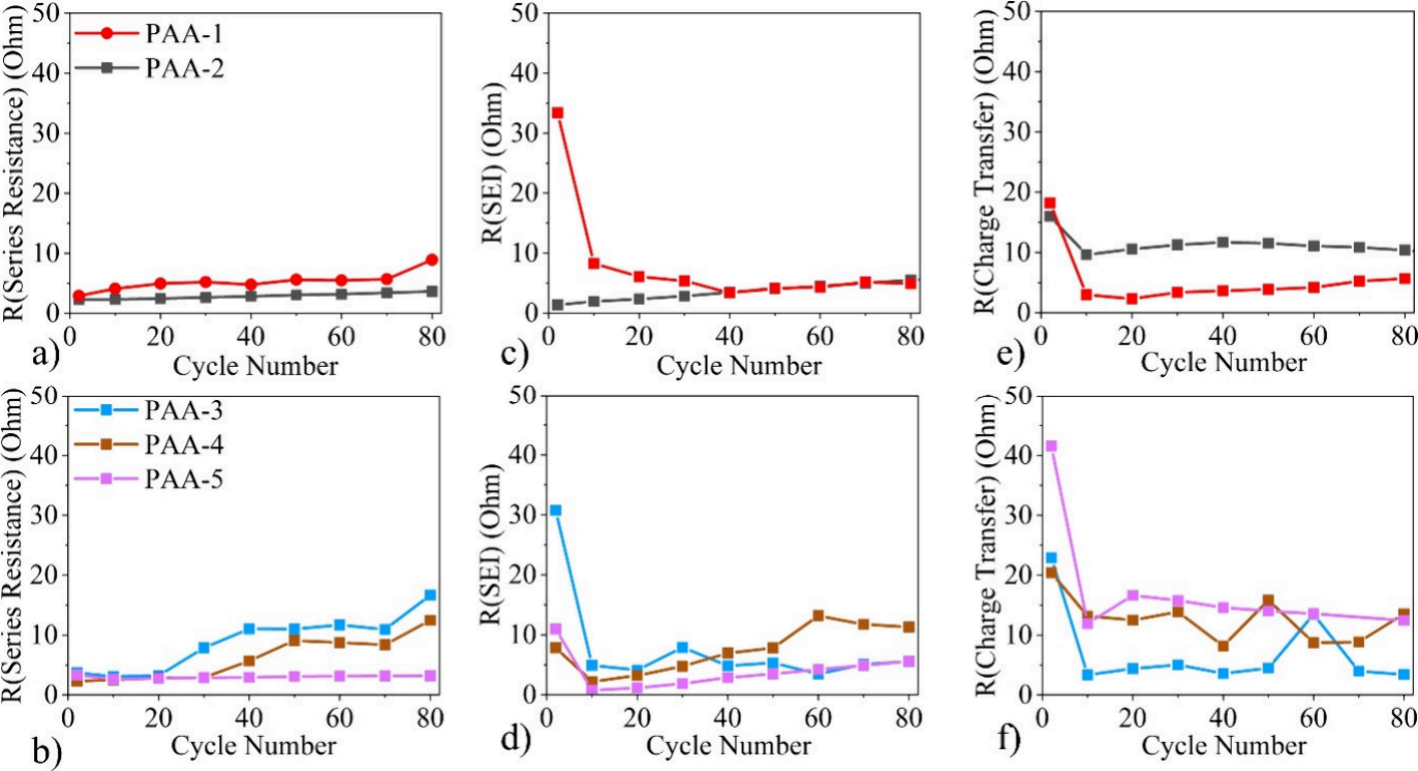
(a, b) Series resistance, (c, d) SEI resistance, and (e, f) charge transfer resistance of the different PAA binder systems during the first 80 cycles.

Figure 7 provides the *R*_S_, *R*_SEI_, and *R*_CT_ for all the systems taken at every 10 cycles for the initial 80 cycles. In Figure 7a,b, both PAA-5 and PAA-2 displayed relatively low *R*_S_ values, which only increased slightly during the 80 cycles. This is a strong indication of good ohmic contact, whereas with the PAA-3 the R_S_ rose sharply by cycles 30 and 40, respectively.^51^ The rise in *R*_S_ for these systems is most likely due to structural degradation within the electrodes, as a result of the silicon’s volume expansion and contraction during cycling.^52^

**Figure 8:**
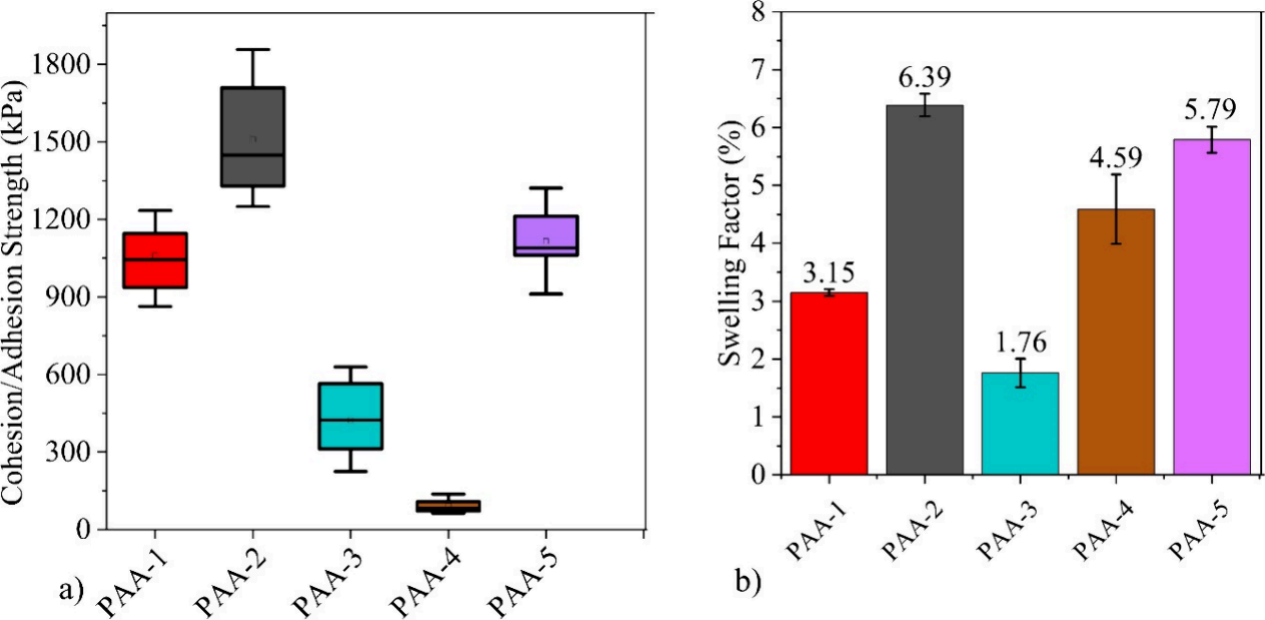
(a) Overview of the cohesion strength of the different PAA binder’s electrode coatings, using Tesa 7262 tape. (b) Overview of polymer electrolyte swelling values after 24 h of polymer being immersed in RD-265 electrolyte.

An increase in *R*_S_ is also linked to a decrease in the electrolyte ionic conductivity, which can be linked to electrode coating delamination. This is potentially the case for PAA-3 and PAA-4, as both systems were found to have cohesion strengths remarkably lower than those of the other PAA systems in Figure 8a, allowing for delamination between the current collector and active material to occur far more easily. Another cause for *R*_S_ to rise is from either changes within the electrolyte composition, or from corrosion of the current collector.^53^ In regards to PAA-4 it is likely to be the former, as their *R*_SEI_ values also increased, and this is attributed to a growing consumption of electrolyte^54^. For PAA-1 and PAA-3 the *R*_SEI_ decreased significantly between cycles 2 to 10. A previous study by J. Liu et al. attributed a similar drop in *R*_SEI_ to the continuous formation and decomposition of an unstable SEI layer.^55^ This can occur if the polymer binder system is unable to prevent the cracking and pulverization of the electrode During this process, silicon’s large volume expansion causes the breakdown of the SEI layer, while a new SEI layer is formed over the freshly exposed surface area of the silicon particles.

The initial *R*_SEI_ values in Figure 7c,d for PAA-2, PAA-4, and PAA-5 were found to be significantly lower than those for PAA 1 and PAA-3. This can be attributed to PAA-2, PAA-4, and PAA-5 being Na-PAAs, in which the extended configuration of their polymer chains allows them to form a homogeneous layer over the surfaces of the active material particles. This polymer layer then acts as an artificial SEI layer which then reduces the consumption of electrolyte during the SEI formation process, resulting in a thinner SEI layer and lower *R*_SEI_ values.^37^ Whereas PAA-1 and PAA-3 are PAAHs, their polymer chains are in an agglomerated state and form a heterogeneous layer instead, in which there are pockets of binder over the surface of the active material. There is then a rougher surface area for forming the SEI layer around it, which results in a thicker SEI layer with more consumption of the electrolyte.

The *R*_SEI_ values in Figure 7c,d were found to sharply decline between cycles 2 and 10 for the bulk of the binder systems, therefore illustrating that the SEI layer had formed. Between cycles 10–80, the *R*_SEI_ values for PAA-5 and PAA-2 were found to only increase gradually, indicating that these systems had formed a relatively stable SEI layer.^56^ By contrast, the initial *R*_SEI_ values for the PAA-3 and PAA-4 systems were found to be slightly higher and rise more rapidly over the 80 cycles compared with those of the other systems. This correlates well with the longer cycling performances (Figure 4a), and thus it can be concluded that the morphological evolution of the SEI layer is a key determining factor in electrode capacity retention.^57^ Furthermore, the heavy influence of binder chemistry on this factor implies that significant gains to cell stability can be achieved through careful selection of the binder material and is to be expected, as it is established that electrodes with higher *R*_SEI_ values experience faster capacity fade.^57^

The *R*_CT_ values are supplied in Figure 7e,f with all systems demonstrating a decline in *R*_CT_ between cycles 2 and 10, attributed to two effects. First, during the initial cycles, the internal pressure of silicon’s large volume expansion results in a more densely packed electrode with improved lithium-ion transport efficiencies.^52^ Second, silicon undergoes a phase transformation from a crystalline to an amorphous state during the initial cycles, which is a more kinetically favorable environment for lithium-ion insertion.^58^ From cycle 10 the *R*_CT_ values of the Na-PAA systems (PAA-2, PAA-4, and PAA-5) were slightly higher than those for the H-PAA systems (PAA-1 and PAA-3). This is possibly due to the carboxylate groups being highly polar, having a strong affinity and a high dissociation energy toward Li^+^ ions.^59^ This potentially makes it more difficult for Li^+^ ions to attach and detach from the carboxylate groups and be transported throughout the active material. As with *R*_S_ and *R*_SEI_, the *R*_CT_ values for PAA-5 and PAA-2 only gradually accumulated over the 80 cycles. As regards to PAA-4, the *R*_CT_ was found to rise sharply from cycle 20 onward. This is another indicator of delamination between the active material and the current collector, and is also possibly the case with PAA-4, as it displayed a poor adhesion strength in Figure 8a.^60^ In addition to this, increases in *R*_CT_ are linked to an electrode undergoing pulverization, hindering lithium ion diffusion and increasing tortuosity through the anode.^61^

Between cycles 40 and 80, the *R*_CT_ values for the PAA-4 and PAA-3 systems were found to undergo periods of rapid increase followed by periods of stabilization. Similar behavior to this has been attributed to be a result of silicon’s large volume expansion as it causes the electrode’s structure to crack, which then results in a variation of the efficiency of Li^+^ transportation (tortuosity) through the active material.^62^ The charge transfer process involves two steps: (i) desolvation of the Li^+^ ions in the electrolyte and (ii) transportation of Li^+^ ions into the active material.^63^ Lower *R*_CT_ values are a result of the electrode becoming cracked as the cracking produces fresh surface areas of the active material, and this allows for electrolyte permeation and easier transportation of lithium ions. However, the *R*_CT_ values also increase again as a new SEI layer is formed over the fresh surface areas, which hinders the transportation of lithium ions. This effect can be correlated with an increase in *R*_SEI_ values, which is the case with the PAA-4 and PAA-3 systems in Figure 7d.

### Cohesion Strength

The cohesion strengths of the electrode coatings were measured using a method reported by W. Haselrieder et al. and were calculated following eq 2.^64^
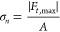
2where σ_*n*_, is the adhesion/cohesion strength, *F*_*t*,max_ is the maximum tensile force and *A* is the surface area of the electrode sample.PAA-1, PAA-2, and PAA-5 had the highest cohesion strengths in Figure 8a, and these binder systems also provided the best electrochemical performances in Figure 4a. This is to be expected because, a higher adhesion strength ensures that there is good electrical contact between the active material and current collector.^65^ First, the cohesion strengths of the high MW PAA-1 and low MW PAA-3 coatings were 1060 and 425 kPa, respectively. This difference is due to PAA-1 containing longer polymer chains with a larger surface area. Because of this, there are more points of interactions between the particles of the active material and polymer binder.^66^

PAA-2 and PAA-5 had the highest cohesion strengths of 1544 and 1115 kPa, respectively. These cohesion strengths were significantly higher than their corresponding non-neutralized counterparts of PAA-1 and PAA-3. This is due to the partial neutralization resulting in the polymer chains being converted from an agglomerated state into an extended state and improving the distribution of the polymer binder throughout the electrode coating. This increases the amount of interactions between the active material and polymer binder.^8^

However, the cohesion strength of the electrode coating only increases up to a certain degree of partial neutralization, as the cohesion strengths of the fully neutralized PAA system (PAA-4) was 89 kPa. This value is also significantly lower than the cohesion strength of the non-neutralized PAA-3 system. As with the cycling performances in Figure 4a, the decreases in adhesion strengths for the fully neutralized systems are again due to the charge screening effect. Here, the excess counterions shield the negatively charged carboxylic groups, which reduces the amount of contact between the polymer chains and the surface of the active material particles.^25^

### Electrolyte Polymer Swelling Tests

Polymer electrolyte swelling tests were performed to investigate the lithium-ion transport efficiencies of the polymer systems. A swelling factor (SW) was used to quantify the degree of swelling and was determined by eq 3.^67^
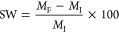
3where *M*_F_ is the mass of the sample following immersion in electrolyte, and *M*_I_ is the mass of the sample before being immersed in electrolyte and the resultant SW for the samples are provided in Figure 8b.The swelling of the polymer binder, along with the porosity within the electrode, is responsible for providing its ionic conductivity.^68^Figure 8b provides the swelling factors of the different binder systems after they were immersed in electrolyte for 24 h.

The MW of the PAA was also found to influence the amount of electrolyte uptake, as the SW of the low MW PAA-3 was only 1.76%, compared to 3.15% for the high Mw PAA-1 system. Surprisingly, this trend disagrees with the findings by A. Magasinski et.al, who found there to be no significant differences between electrolyte swelling and MW.^6^ Overall, the values obtained agree with the findings from previous studies, which also reported low levels of electrolyte swelling with PAA based binder systems.^69^ The reason for this is that the polar–polar interactions between the polymer chains are more favorable and desirable than the polar-nonpolar interactions between the polymer chains and electrolyte molecules.^70^

Partial and fully neutralized PAA systems were found to have higher electrolyte uptake values compared to non-neutralized PAA. For the other low MW systems, the SW values were 5.79% and 4.59% for the PAA-5 and PAA-4, respectively. A similar trend was established with the high MW samples, with the SW for PAA-1 being 3.15% and 6.39% for the PAA-2. These improved swelling factors are due to the ionized carboxylate groups present in the partial and fully neutralized PAA systems.^71^

However, as with the cohesion strengths, it is only up to a certain degree of partial neutralization that an increase in electrolyte uptake is observed. For example, the PAA-4 system has a greater degree of neutralization than the PAA-5 system, and yet it has a slightly lower SW value. In PAA it is expected for the SW to reach a maximum at around pH 7, before again decreasing at higher pHs.^72^ Again, this is primarily due to the charge screening effect that occurs at higher pH. The charge screening effect decreases the amount of repulsions between the polymer chains, and hence reduces the space between the polymer chains for accommodating solvent electrolyte molecules.^25^ The systems with the highest SW values (PAA-2 and PAA-5) provided the best electrochemical performances. These higher SW values indicate a high lithium-ion transport efficiency for these binder systems, which is important for achieving the full capacity of the overall electrode. This is due to the binder being dispersed throughout the electrode, and a lower lithium ion efficiency in the binder could result in impeding lithium ion transport.^64^

### Rate Capability Tests

**Figure 9:**
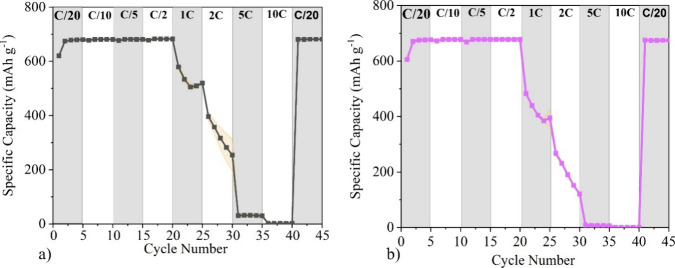
Rate capability tests for (a) PAA-2 and (b) PAA-5 systems at C-rates of C/20, C/10, C/5, C/2, 1C, 2C, 5C, 10C and C/20.

Rate capability tests were performed on the two best performing PAA binder systems (PAA-2 and PAA-5) to determine if the difference in their MW would provide a more favorable environment for lithium-ion kinetics. Both systems were charged at the following C-rates; C/20, C/10, C/5, 1C, 2C, 5C and 10C, as shown in Figure 9. Stable capacities were observed for both systems at lower C-rates (C/20, C/10, C/5, and C/2). Meanwhile at the higher C-rates (1C, 2C, 5C and 10C) the capacity of both systems was found to have dropped significantly. Slightly higher capacities were observed for the PAA-2 system during these C-rates. For example, at 5C the average battery capacity retention for PAA-2 was 30 mAh g^–1^, compared to only 6 mAh g^–1^ for the PAA-5 system. This small difference is most likely due to the slightly lower electrode mass loading of PAA-2 compared to PAA-5 as shown in Table 3. A higher mass loading will result in a slightly thicker electrode and as such there will be a greater resistance associated with transporting the lithium ions throughout it.

Once the C-rate was restored to C/20, the capacities of both PAA-2 and PAA-5 were found to return to their original levels, indicating that the capacity decline experienced at the higher C-rates was not irreversible. The similar trend observed between PAA-2 and PAA-5 suggests that this behavior is independent of the MW of the binder systems and that both binder systems were able to effectively maintain the electrodes’ microstructure during the silicon’s volume expansion. Instead, the significant drop in capacity at the higher C-rates is most likely due to the conductivity of the silicon/graphite anode system overall not being optimal.^73^ Silicon is reported to have poor ionic conductivity properties and slow lithium-ion diffusion kinetics with sluggish lithium-ion diffusion coefficients that range from 10^–14^–10^–13^ cm^2^ s^–1^, heavily hindering its rate capability.^74^

**Figure 10:**
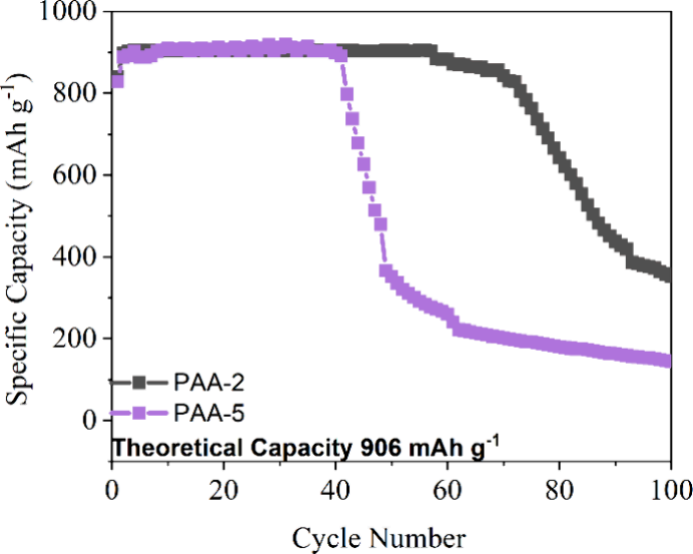
Cycling performance for PAA-2 and PAA-5 at a theoretical capacity of 906 mAh g^–1^.

**Figure 11:**
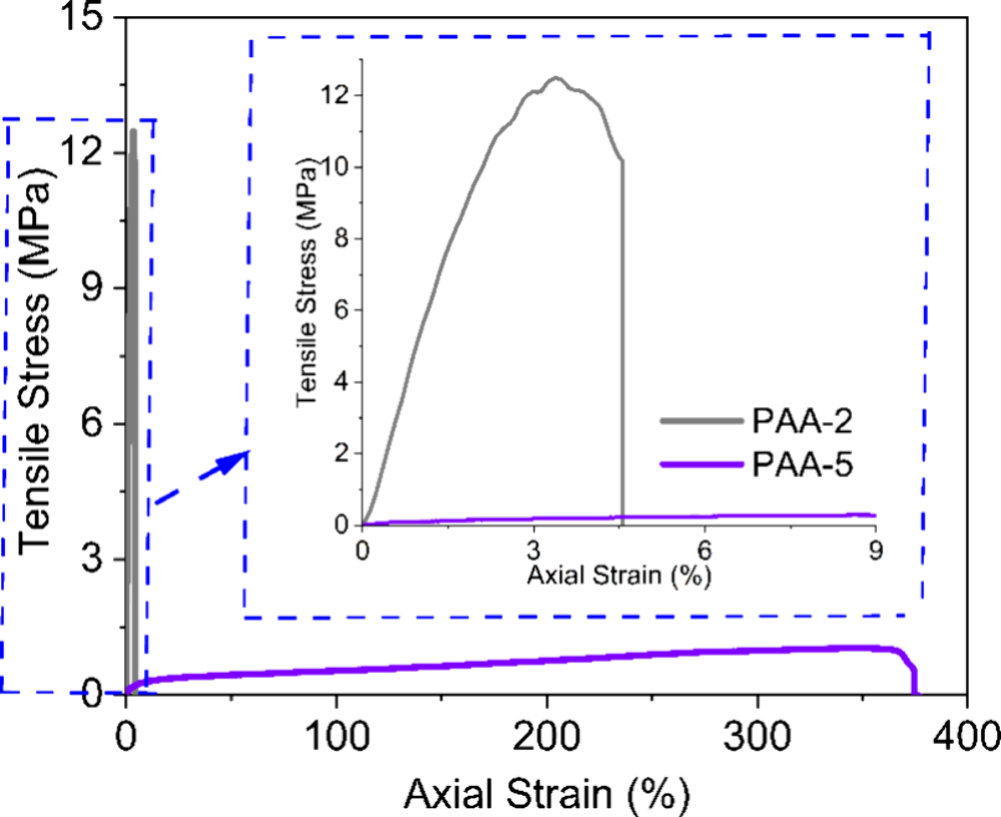
Stress strain curves for PAA-2 and PAA-5.

The electrochemical performances of PAA-2 and PAA-5 were re-evaluated, with the theoretical capacity limit of the silicon being increased to 1800 mAh g^–1^. Due to the electrode’s formulation consisting of 50% graphite, the overall theoretical capacities of the cells were 906 mAh g-^1^ respectively. Figure 10 provides these cycling data, showing that PAA-2 and PAA-5 had stable capacity retentions for only 35 and 60 cycles, respectively. Yet again this shows that the high MW Na-PAA binder system has a more stable capacity retention and therefore is better at maintaining the electrodes microstructure during the volume expansion. The differences in the performances of the MW can be explained through their mechanical properties in Figure 11.

### Tensile Testing

Polymer films of the two best electrochemically performing PAAs (PAA-2 and PAA-5) were prepared as described in the methodology section, and their mechanical properties were investigated via tensile testing. The MW was found to heavily influence the mechanical properties of the PAA samples as shown in the resultant stress strain curves in Figure 11 and summarized in Table 5. The high MW PAA-2 sample, displayed typical linear stress–strain behavior without plastic deformation, agreeing with the mechanical properties of Na-PAA reported in previous studies.^75^ The low MW PAA-5 sample on the other hand demonstrated typical behavior corresponding to elastomer based polymers.^76^ Testing revealed the PAA-2 system to have a Young’s modulus value of 803 MPa and a strain at failure of 13.41%. For the PAA-5 the Young’s modulus value obtained was 3.75 MPa, while the strain at failure was 376.99%.

**Table 5:** Overview of PAA-2 and PAA-5 Tensile Testing Properties^a^

binder system	Young’s modulus (MPa)	strain at failure (%)	stress at failure (MPa)
PAA-2	803.00 ± 126.35	13.41 ± 1.51	31.03 ± 1.08
PAA-5	3.75 ± 1.74	376.99 ± 19.73	1.01 ± 0.16

a ± refers to the standard deviation based on a minimum of three specimens.

PAA-5 is a low MW polymer and, as such, possesses a relatively low ultimate tensile strength. This is due to its polymer chains being only loosely held together via intermolecular interactions, meaning they can more easily slide over each other. Meanwhile the PAA-2 system is a high MW polymer, which contains significantly longer polymer chains, and therefore, there is a greater possibility of the chains becoming entangled. This makes it more difficult for the chains to slide over each other and hence the PAA-2 has a much higher ultimate tensile strength than PAA-5.^77^ Some studies state that binder systems with high flexibilities and good tensile strain’s as the case with PAA-5, make better candidates for assisting in maintaining the electrodes microstructure during the volume expansion.^78^ However, other studies as in the case here with PAA-2, state that binders systems with a high Young’s Modulus value provides the silicon/graphite anode with the mechanical stability required for buffering the volume expansion.^79^ Hence the high MW PAA-2 system still had a longer capacity retention at the higher capacity limit in Figure 10.

## Conclusion

In this study, low MW Na-PAAs at different degrees of neutralization were explored as possible binder systems for the application of silicon/graphite anodes. There performances were compared directly to their pure PAA counterparts, and the best system was found to be a high MW 70% partially neutralized Na-PAA. Meanwhile, the poorest performing PAA system was established to be the low MW PAA system. The improved performance of high MW Na-PAA was established because of the following factors. First, the partial neutralization results in an improved distribution of the binder system throughout the electrode coating due to the polymer chain configuration being converted from a coil to an extended state. Second, this results in these systems having far greater adhesion and cohesion strengths. This is because the extended polymer chain configuration causes there to be far more points of contact between the active material and the polymer binder. Thus, it is highly likely that these binder systems are more effective in maintaining the electrodes’ microstructure during cycling. Third, the longer polymer chains found within the higher MW Na-PAA polymer chains have a greater chance of becoming entangled and therefore also providing the electrode with the necessary mechanical stability. These factors result in overall improved capacity retentions in silicon graphite anodes.

## References

[ref1] Zhang Z.; Han X.; Li L.; Su P.; Huang W.; Wang J.; Xu J.; Li C.; Chen S.; Yang Y. Tailoring the interfaces of silicon/carbon nanotube for high rate lithium-ion battery anodes. J. Power Sources 2020, 450, 227593 10.1016/j.jpowsour.2019.227593.

[ref2] Hamzelui N.; Eshetu G. G.; Figgemeier E. Customizing Active Materials and Polymeric Binders: Stern Requirements to Realize Silicon-Graphite Anode Based Lithium-Ion Batteries. J. Energy Storage 2021, 35, 102098 10.1016/j.est.2020.102098.

[ref3] Wang Z.; Zheng B.; Liu H.; Zhang C.; Wu F.; Luo H.; Yu P. One-step synthesis of nanoporous silicon @ graphitized carbon composite and its superior lithium storage properties. J. Alloys Compd. 2021, 861, 157955 10.1016/j.jallcom.2020.157955.

[ref4] Wang S.; Duan Q.; Lei J.; Yu D. Y. W. Slime-inspired polyacrylic acid-borax crosslinked binder for high-capacity bulk silicon anodes in lithium-ion batteries. J. Power Sources 2020, 468, 228365 10.1016/j.jpowsour.2020.228365.

[ref5] Zhu L.; Chen Y.; Wu C.; Chu R.; Zhang J.; Jiang H.; Zeng Y.; Zhang Y.; Guo H. Double-carbon protected silicon anode for high performance lithiumion batteries. J. Alloys Compd. 2020, 812, 151848 10.1016/j.jallcom.2019.151848.

[ref6] Magasinski A.; Zdyrko B.; Kovalenko I.; Hertzberg B.; Burtovyy R.; Huebner C. F.; Fuller T. F.; Luzinov I.; Yushin G. Toward Efficient Binders for Li-Ion Battery Si-Based Anodes: Polyacrylic Acid. ACS Appl. Mater. Interfaces 2010, 2 (11), 3004–3010. 10.1021/am100871y. 21053920

[ref7] Jolley M. J.; Pathan T. S.; Wemyss A. M.; Prokes I.; Moharana S.; Wan C.; Loveridge M. J. Development and Application of a Poly(acrylic acid)-Grafted Styrene–Butadiene Rubber as a Binder System for Silicon-Graphite Anodes in Li-Ion Batteries. ACS Appl. Energy Mater. 2023, 6, 496–507. 10.1021/acsaem.2c03489.

[ref8] Huang Q.; Wan C.; Loveridge M.; Bhagat R. Partially Neutralized Polyacrylic Acid/Poly(vinyl alcohol) Blends as Effective Binders for High-Performance Silicon Anodes in LithiumIon Batteries. ACS Appl. Energy Mater. 2018, 1, 6890–6898. 10.1021/acsaem.8b01277.

[ref9] Chuang Y.; Lin Y.; Wang C.; Hong J. Dual Cross-Linked Polymer Networks Derived from the Hyperbranched Poly(ethyleneimine) and Poly(acrylic acid) as Efficient Binders for Silicon Anodes in Lithium-Ion Batteries. ACS Appl. Energy Mater. 2021, 4, 1583–1592. 10.1021/acsaem.0c02802.

[ref10] Hays K. A.; Ruther R. E.; Kukay A. J.; Cao P.; Saito T.; Wood D. L.; Li J. What makes lithium substituted polyacrylic acid a better binder than polyacrylic acid for silicon-graphite composite anodes?. J. Power Sources 2018, 384, 136–144. 10.1016/j.jpowsour.2018.02.085.

[ref11] Ma D.; Cao Z.; Hu A. Si-Based Anode Materials for Li-Ion Batteries: A Mini Review. Nano-Micro Lett. 2014, 6, 347–358. 10.1007/s40820-014-0008-2. PMC6223966 30464946

[ref12] Liu L.; Luo S.; Wang B.; Guo Z. Investigation of small molecular weight poly(acrylic acid) adsorption on γ-alumina. Applied Surface Scince 2015, 345, 116–121. 10.1016/j.apsusc.2015.03.145.

[ref13] Zhang S.; Liu K.; Xie J.; Xu X.; Tu J.; Chen W.; Chen F.; Zhu T.; Zhao X. An Elastic Cross-Linked Binder for Silicon Anodes in Lithium-Ion Batteries with a High Mass Loading. ACS Appl. Mater. Interfaces 2023, 15, 6594–6602. 10.1021/acsami.2c16997. 36705634

[ref14] Liang S.; Zhang J.; Jia C.; Luo Z.; Zhang L. Water soluble polymer binder with good mechanical property and ionic conductivity for high performance lithium sulfur battery. Carbon 2024, 222, 118807 10.1016/j.carbon.2024.118807.

[ref15] Kasinathan R.; Marinaro M.; Axmann P.; Wohlfahrt-Mehrens M. Influence of the Molecular Weight of Poly-Acrylic Acid Binder on Performance of Si-Alloy/Graphite Composite Anodes for Lithium-Ion Batteries. Energy Technol. 2018, 6, 2256–2263. 10.1002/ente.201800302. PMC6358114 30775217

[ref16] Xie R.; Weisen A. R.; Lee Y.; Aplan M. A.; Fenton A. M.; Masucci A. E.; Kempe F.; Sommer M.; Pester C. W.; Colby R. H.; Gomez E. D. Glass transition temperature from the chemical structure of conjugated polymers. Nat. Commun. 2020, 11, 1–20. 10.1038/s41467-020-14656-8. 32060331 PMC7021822

[ref17] Assresahegn B. D.; Ossonon B. D.; Bélanger D. Graphene nanosheets and polyacrylic acid grafted silicon composite anode for lithium ion batteries. J. Power Sources 2018, 391, 41–50. 10.1016/j.jpowsour.2018.03.067.

[ref18] Asgreen C.; Knopp M. M.; Skytte J.; Löbmann K. Influence of the Polymer Glass Transition Temperature and Molecular Weight on Drug Amorphization Kinetics Using Ball Milling. Pharmceutics 2020, 12, 483 10.3390/pharmaceutics12060483. PMC7355600 32471023

[ref19] Hu B.; Shkrob I. A.; Zhang S.; Zhang L.; Zhang J.; Li Y.; Liao C.; Zhang Z.; Lu W.; Zhang L. The existence of optimal molecular weight for poly(acrylic acid) binders in silicon/graphite composite anode for lithium-ion batteries. J. Power Sources 2018, 378, 671–675. 10.1016/j.jpowsour.2017.12.068.

[ref20] Armstrong B. L.; Hays K. A.; Ruther R. E.; Hawley W. B.; Rogers A.; Greeley I.; Cavallaro K. A.; Veith G. M. Role of silicon-graphite homogeneity as promoted by low molecular weight dispersants. J. Power Sources 2022, 517, 230671 10.1016/j.jpowsour.2021.230671.

[ref21] Han Z.-J.; Yamagiwa K.; Yabuuchi N.; Son J.-Y.; Cui Y.-T.; Oji H.; Kogure A.; Harada T.; Ishikawa S.; Aoki Y.; Komaba S. Electrochemical lithiation performance and characterization of silicon–graphite composites with lithium, sodium, potassium, and ammonium polyacrylate binders. Phys. Chem. Chem. Phys. 2015, 17, 3783–3795. 10.1039/C4CP04939J. 25559330

[ref22] Dubinsky S.; Grader G. S.; Shter G. E.; Silverstein M. S. Thermal degradation of poly(acrylic acid) containing copper nitrate. Polym. Degrad. Stab. 2004, 86, 171–178. 10.1016/j.polymdegradstab.2004.04.009.

[ref23] Yuan J.; Sun C. C.; Fang L.; Song Y.; Yan Y.; Qiu Z.; Shen Y.; Li H.; Zhu B. A lithiated gel polymer electrolyte with superior interfacial performance for safe and long-life lithium metal battery. J. Energy Chem. 2021, 55, 313–322. 10.1016/j.jechem.2020.06.052.

[ref24] Sheridan J.; Sonebi M.; Taylor S.; Amziane S. The effect of a polyacrylic acid viscosity modifying agent on the mechanical, thermal and transport properties of hemp and rapeseed straw concrete. Constr. Build. Mater. 2020, 235, 117536 10.1016/j.conbuildmat.2019.117536.

[ref25] Yang F.; Ma J.; Bao Y.; Zhu Q.; Zhang W. Swelling Behaviors of Super-Absorbent Hydrogel based Waste: Bacteria Bran. J. Environ. Polym. Degrad 2021, 29, 1542–1550. 10.1007/s10924-020-01948-9.

[ref26] Li Q.; Yuan R.; Li Y. Study on the Molecular Behavior of Hydrophobically Modified Poly(acrylic acid) in Aqueous Solution and Its Emulsion-Stabilizing Capacity. J. Appl. Polym. Sci. Rep. 2013, 128, 206–215. 10.1002/app.38169.

[ref27] Wang Y.; Wang X.; Li X.; Bai Y.; Xiao H.; Liu Y.; Yuan G. Scalable fabrication of polyaniline nanodots decorated MXene film electrodes enabled by viscous functional inks for high-energy-density asymmetric supercapacitors. Chem. Eng. Sci. 2021, 405, 126664 10.1016/j.cej.2020.126664.

[ref28] Ouyang L.; Wu Z.; Wang J.; Qi X.; Li Q.; Wang J.; Lu S. The effect of solid content on the rheological properties and microstructures of a Li-ion battery cathode slurry. RSC Adv. 2020, 10, 19360 10.1039/D0RA02651D. 35515438 PMC9054056

[ref29] Huang Q.; Loveridge M. J.; Genieser R.; Lain M. J.; Bhagat R. Electrochemical Evaluation and Phase-related Impedance Studies on Silicon–Few Layer Graphene (FLG) Composite Electrode Systems. Sci. Rep. 2018, 8 (1386), 1–9. 10.1038/s41598-018-19929-3. 29362384 PMC5780504

[ref30] Yang X.; Zhan P.; Wen Z.; Zhang L. High performance silicon/carbon composite prepared by in situ carbon-thermal reduction for lithium ion batteries. J. Alloys Compd. 2010, 496, 403–406. 10.1016/j.jallcom.2010.02.035.

[ref31] Lin J.; Xu Y.; Wang J.; Zhang B.; Wang C.; He S.; Wu J. Preinserted Li metal porous carbon nanotubes with high Coulombic efficiency for lithium-ion battery anodes. Chem. Eng. J. 2019, 373, 78–85. 10.1016/j.cej.2019.04.204.

[ref32] Schulze M. C.; Neale N. R. Half-Cell Cumulative Efficiency Forecasts FullCell Capacity Retention in Lithium-Ion Batteries. ACS Energy Lett. 2021, 6, 1082–1086. 10.1021/acsenergylett.1c00173.

[ref33] Azeemi R. Y.; Ergün R.; Taşdemir A.; Gürsel S. A.; Yürüm A. Y. A simple spray assisted method to fabricate high performance layered graphene/silicon hybrid anodes for lithium-ion batteries. Int. J. Hydrogen Energy 2019, 44, 20267–20277. 10.1016/j.ijhydene.2019.05.200.

[ref34] Cui X.; Geng T.; Zhang F.; Zhang N.; Zhao D.; Li C.; Li S. The influence of the voltage plateau on the coulombic efficiency and capacity degradation in LiNi_0.5_Mn_1.5_O_4_ materials. J. Alloys Compd. 2020, 820, 153443 10.1016/j.jallcom.2019.153443.

[ref35] Tao Y.; Wei C.; Fei H.; An Y.; Tian Y.; Wei H.; Xiong S.; Feng J. Controlled synthesis of copper reinforced nanoporous silicon microsphere with boosted electrochemical performance. J. Power Sources 2020, 455, 227967 10.1016/j.jpowsour.2020.227967.

[ref36] Wei L.; Chen C.; Hou Z.; Wei H. Poly (acrylic acid sodium) grafted carboxymethyl cellulose as a high performance polymer binder for silicon anode in lithium ion batteries. Sci. Rep. 2016, 6, 1–8. 10.1038/srep19583. 26786315 PMC4726210

[ref37] Xiong J.; Dupré N.; Mazouzi D.; Guyomard D.; Roué L.; Lestriez B. Influence of the Polyacrylic Acid Binder Neutralization Degree on the Initial Electrochemical Behavior of a Silicon/Graphite Electrode. ACS Appl. Mater. Interfaces 2021, 13, 28304–28323. 10.1021/acsami.1c06683. 34101424

[ref38] Komaba S.; Okushi K.; Ozeki T.; Yui H.; Katayama Y.; Miura T.; Saito T.; Groult H. Polyacrylate Modifier for Graphite Anode of Lithium-Ion Batteries. Electrochem. Solid-State Lett. 2009, 12, A107–A110. 10.1149/1.3086262.

[ref39] Porcher W.; Chazelle S.; Boulineau A.; Mariage N.; Alper J. P.; Van Rompaey T.; Bridel J.-S.; Haon C. Understanding Polyacrylic Acid and Lithium Polyacrylate Binder Behavior in Silicon Based Electrodes for Li-Ion Batteries. J. Electrochem. Soc. 2017, 164, A3633–A3640. 10.1149/2.0821714jes.

[ref40] Son S.; Cao L.; Yoon T.; Cresce A.; Hafner S. E.; Liu J.; Groner M.; Xu K.; Ban C. Interfacially Induced Cascading Failure in Graphite-Silicon Composite Anodes. Adv. Sci. 2019, 6, 1801007 10.1002/advs.201801007. PMC6364491 30775222

[ref41] Azib T.; Thaury C.; Fariaut-Georges C.; Hézèque T.; Cuevas F.; Jordy C.; Latroche M. Role of silicon and carbon on the structural and electrochemical properties of Si-Ni_3.4_Sn_4_-Al-C anodes for Li-ion batteries. Mater. Today Commun. 2020, 23, 101160 10.1016/j.mtcomm.2020.101160.

[ref42] Nguyen C. C.; Yoon T.; Seo D. M.; Guduru P.; Lucht B. L. Systematic Investigation of Binders for Silicon Anodes: Interactions of Binder with Silicon Particles and Electrolytes and Effects of Binders on Solid Electrolyte Interphase Formation. ACS Appl. Mater. Interfaces 2016, 8, 12211–12220. 10.1021/acsami.6b03357. 27135935

[ref43] Chen D.; Yi R.; Chen S.; Xu T.; Gordin M. L.; Wang D. Facile synthesis of graphene–silicon nanocomposites with an advanced binder for high-performance lithium-ion battery anodes. Solid State Ion 2014, 254, 65–71. 10.1016/j.ssi.2013.11.020.

[ref44] Ulvestad A.; Reksten A. H.; Andersen H. F.; Carvalho P. A.; Jensen I. J. T.; Nagell M. U.; Mæhlen J. P.; Kirkengen M.; Koposov A. Y. Crystallinity of Silicon Nanoparticles: Direct Influence on the Electrochemical Performance of Lithium Ion Battery Anodes. ChemElectroChem 2020, 7, 4349–4353. 10.1002/celc.202001108.

[ref45] Hoeltgen C.; Lee J.; Jang B. Stepwise carbon growth on Si/SiO_x_ core-shell nanoparticles and its effects on the microstructures and electrochemical properties for high-performance lithium-ion battery’s anode. Electrochim. Acta 2016, 222, 535–542. 10.1016/j.electacta.2016.11.006.

[ref46] Stetson C.; Yin Y.; Norman A.; Harvey S. P.; Schnabel M.; Ban C.; Jiang C.; DeCaluwe S. C.; Al-Jassim M. Evolution of solid electrolyte interphase and active material in the silicon wafer model system. J. Power Sources 2021, 482, 228946 10.1016/j.jpowsour.2020.228946.

[ref47] Choi J.; Kim H.; Jin E.; Seo M. W.; Cho J. S.; Kumar R. V.; Jeong S. M. Facile and scalable synthesis of silicon nanowires from waste rice husk silica by the molten salt process. J. Hazard. Mater. 2020, 399, 122949 10.1016/j.jhazmat.2020.122949. 32502856

[ref48] Wetjen M.; Pritzl D.; Jung R.; Solchenbach S.; Ghadimi R.; Gasteiger H. A. Differentiating the Degradation Phenomena in Silicon-Graphite Electrodes for Lithium-Ion Batteries. J. Electrochem. Soc. 2017, 164, A2840–A2852. 10.1149/2.1921712jes.

[ref49] Lv Q.; Liu Y.; Ma T.; Zhu W.; Qiu X. Hollow Structured Silicon Anodes with Stabilized Solid Electrolyte Interphase Film for Lithium-Ion Batteries. ACS Appl. Mater. Interfaces 2015, 7, 23501–23506. 10.1021/acsami.5b05970. 26402521

[ref50] Guo R.; Zhang S.; Ying H.; Yang W.; Wang J.; Han W. New, Effective, and Low-Cost Dual-Functional Binder for Porous Silicon Anodes in Lithium-Ion Batteries. ACS Appl. Mater. Interfaces 2019, 11, 14051–14058. 10.1021/acsami.8b21936. 30901188

[ref51] Li B.; Zhao W.; Yang Z.; Zhang C.; Dang F.; Liu Y.; Jin F.; Chen X. A carbon-doped anatase TiO_2_-Based flexible silicon anode with high-performance and stability for flexible lithium-ion battery. J. Power Sources 2020, 466, 228339 10.1016/j.jpowsour.2020.228339.

[ref52] Malik R.; Huang Q.; Silvestri L.; Liu D.; Pellegrini V.; Marasco L.; Venezia E.; Abouali S.; Bonaccorso F.; Lain M. J.; Greenwood D.; West G.; Shearing P. R.; Loveridge M. J. Synthesis of layered silicon-graphene hetero-structures by wet jet milling for high capacity anodes in Li-ion batteries. 2D Mater. 2021, 8, 015012 10.1088/2053-1583/aba5ca.

[ref53] Selvapandiyan M.; Balaji G.; Sivakumar N.; Prasath M.; Sagadevan S. Influence of pomegranate inclusion towards the electrochemical performance of lithium hexafluorophosphate in lithium-ion batteries. Chem. Phys. Lett. 2021, 762, 138118 10.1016/j.cplett.2020.138118.

[ref54] Carter R.; Kingston T. A.; Atkinson R. W.; Parmananda M.; Dubarry M.; Fear C.; Mukherjee P. P.; Love C. T. Directionality of thermal gradients in lithiumion batteries dictates diverging degradation modes. Cell Rep. Phys. Sci. 2021, 2, 100351 10.1016/j.xcrp.2021.100351.

[ref55] Liu J.; Li C.; Dong B.; Yan Y.; Zerrin T.; Ozkan M.; Ozkan C. S. Scalable coral-like silicon powders with three-dimensional interconnected structures for lithium ion battery anodes. Energy Storage 2020, 2, e187 10.1002/est2.187.

[ref56] Jiang S.; Hu B.; Sahore R.; Zhang L.; Liu H.; Zhang L.; Lu W.; Zhao B.; Zhang Z. Surface-Functionalized Silicon Nanoparticles as Anode Material for Lithium-Ion Battery. ACS Appl. Mater. Interfaces 2018, 10, 44924–44931. 10.1021/acsami.8b17729. 30485060

[ref57] Yu L.; Liu J.; He S.; Huang C.; Gan L.; Gong Z.; Long M. A novel high-performance 3D polymer binder for silicon anode in lithiumion batteries. J. Phys. Chem. Solids 2019, 135, 109113 10.1016/j.jpcs.2019.109113.

[ref58] Guo J.; Omar A.; Urbanski A.; Oswald S.; Uhlmann P.; Giebeler L. Electrochemical Behavior of Microparticulate Silicon Anodes in Ether-Based Electrolytes: Why Does LiNO3 Affect Negatively?. ACS Appl. Energy Mater. 2019, 2, 4411–4420. 10.1021/acsaem.9b00590.

[ref59] Zhao Y.; Liang Z.; Kang Y.; Zhou Y.; Li Y.; He X.; Wang L.; Mai W.; Wang X.; Zhou G.; Wang J.; Li J.; Tavajohi N.; Li B. Rational design of functional binder systems for high-energy lithium-based rechargeable batteries. Energy Storage Mater. 2021, 35, 353–377. 10.1016/j.ensm.2020.11.021.

[ref60] Guo J.; Sun A.; Chen X.; Wang C.; Manivannan A. Cyclability study of silicon–carbon composite anodes for lithium-ion batteries using electrochemical impedance spectroscopy. Electrochim. Acta 2011, 56, 3981–3987. 10.1016/j.electacta.2011.02.014.

[ref61] Chen C.; Chen F.; Liu L.; Zhao J.; Wang F. Cross-linked hyperbranched polyethylenimine as an efficient multidimensional binder for silicon anodes in lithium-ion batteries. Electrochim. Acta 2019, 326, 134964 10.1016/j.electacta.2019.134964.

[ref62] Malik R.; Loveridge M. J.; Williams L. J.; Huang Q.; West G.; Shearing P. R.; Bhagat R.; Walton R. I. Porous Metal–Organic Frameworks for Enhanced Performance Silicon Anodes in Lithium-Ion Batteries. Chem. Mater. 2019, 31, 4156–4165. 10.1021/acs.chemmater.9b00933.

[ref63] Jow T. R.; Delp S. A.; Allen J. L.; Jones J.; Smart M. C. Factors Limiting Li^+^ Charge Transfer Kinetics in Li-Ion Batteries. J. Electrochem. Soc. 2018, 165, A361–A367. 10.1149/2.1221802jes.

[ref64] Haselrieder W.; Westphal B.; Bockholt H.; Diener A.; Höft S.; Kwade A. Measuring the coating adhesion strength of electrodes for lithium-ion batteries. Int. J. Adhes Adhes 2015, 60, 1–8. 10.1016/j.ijadhadh.2015.03.002.

[ref65] Jenkins C. A.; Coles S. R.; Loveridge M. J. Investigation into Durable Polymers with Enhanced Toughness and Elasticity for Application in Flexible Li-Ion Batteries. ACS Appl. Energy Mater. 2020, 3, 12494–12505. 10.1021/acsaem.0c02442.

[ref66] Park H.; Kong B.; Oh E. Effect of high adhesive polyvinyl alcohol binder on the anodes of lithium ion batteries. Electrochem. commun. 2011, 13, 1051–1053. 10.1016/j.elecom.2011.06.034.

[ref67] Jolley M. J.; Pathan T. S.; Jenkins C.; Loveridge M. J. Investigating the effect of the degree of cross-linking instyrene butadiene rubber on the performance of graphite anodes for the use in lithium-ion batteries. J. Appl. Polym. Sci. 2024, 141, e55135 10.1002/app.55135.

[ref68] Zhao H.; Yuan W.; Liu G. Hierarchical electrode design of high-capacity alloy nanomaterials for lithium-ion batteries. Nano Today 2015, 10, 193–212. 10.1016/j.nantod.2015.02.009.

[ref69] Urbanski A.; Omar A.; Guo J.; Janke A.; Reuter U.; Malanin M.; Schmidt F.; Jehnichen D.; Holzschuh M.; Simon F.; et al. An Efficient Two-Polymer Binder for High-Performance Silicon Nanoparticle-Based Lithium-Ion Batteries: A Systematic Case Study with Commercial Polyacrylic Acid and Polyvinyl Butyral Polymers. J. Electrochem. Soc. 2019, 166, A5275–A5286. 10.1149/2.0371903jes.

[ref70] Nguyen Q. D.; Oh E.; Chung K. Nanomechanical properties of polymer binders for Li-ion batteries probed with colloidal probe atomic force microscopy. Polym. Test. 2019, 76, 245–253. 10.1016/j.polymertesting.2019.03.025.

[ref71] Yuca N.; ÇOlak Ü. A facile and functional process to enhance electrochemical performance of silicon anode in lithium ion batteries. Electrochim. Acta 2016, 222, 1538–1544. 10.1016/j.electacta.2016.11.136.

[ref72] Lim L. S.; Ahmad I.; Lazim A. M. pH Sensitive Hydrogel Based on Poly(Acrylic Acid) and Cellulose Nanocrystals. Sains Malaysiana 2015, 44, 779–785. 10.17576/jsm-2015-4406-02.

[ref73] Shi M.; Song C.; Tai Z.; Zou K.; Duan Y.; Dai X.; Sun J.; Chen Y.; Liu Y. Coal-derived synthetic graphite with high specific capacity and excellent cyclic stability as anode material for lithium-ion batteries. Fuel 2021, 292, 120250 10.1016/j.fuel.2021.120250.

[ref74] Liu X.; Zhu X.; Pan D. Solutions for the problems of silicon–carbon anode materials for lithium-ion batteries. R. Soc. open sci. 2018, 5, 172370 10.1098/rsos.172370. 30110426 PMC6030270

[ref75] Wang S.; Liu D.; Cai X.; Zhang L.; Liu Y.; Qin X.; Zhao R.; Zeng X.; Han C.; Zhan C.; Kang F.; Li B. Promoting the reversibility of lithium ion/lithium metal hybrid graphite anode by regulating solid electrolyte interface. Nano Energy 2021, 90, 106510 10.1016/j.nanoen.2021.106510.

[ref76] Luo Y.; Sun L.; Xu F.; Wei S.; Wang Q.; Peng H.; Chen C. Cobalt(II) coordination polymers as anodes for lithium-ion batteries with enhanced capacity and cycling stability. J. Mater. Sci. Technol. 2018, 34, 1412–1418. 10.1016/j.jmst.2017.11.006.

[ref77] Balani K.; Verma V.; Agarwal A.; Narayan R. Physical, Thermal, and Mechanical Properties of Polymers. In Biosurfaces: A Materials Science and Engineering Perspective, 1st ed; Balani K., Verma V., Agarwal A., Narayan R., Eds.; John Wiley & Sons, 2015, Chapter A1, p 337.

[ref78] Deng L.; Deng S.-S.; Pan S.-Y.; Wu Z.-Y.; Hu Y.-Y.; Li K.; Zhou Y.; Li J.-T.; Huang L.; Sun S.-G. Multivalent Amide-Hydrogen-Bond Supramolecular Binder Enhances the Cyclic Stability of Silicon-Based Anodes for Lithium-Ion Batteries. ACS Appl. Mater. Interfaces 2021, 13 (19), 22567–22576. 10.1021/acsami.1c04501. 33945259

[ref79] Wei L.; Hou Z. High performance polymer binders inspired by chemical finishing of textiles for silicon anodes in lithium ion batteries. J. Mater. Chem. A 2017, 5, 22156–22162. 10.1039/C7TA05195F.

